# Test-Retest Reliability of Diffusion Tensor Imaging in Huntington’s Disease

**DOI:** 10.1371/currents.hd.f19ef63fff962f5cd9c0e88f4844f43b

**Published:** 2014-03-21

**Authors:** James H. Cole, Ruth E. Farmer, Elin M. Rees, Hans J. Johnson, Chris Frost, Rachael I. Scahill, Nicola Z. Hobbs

**Affiliations:** Huntington's Disease Research Group, Department of Neurodegenerative Disease, UCL Institute of Neurology, London, UK; Computational, Cognitive & Clinical Neuroimaging Laboratory, Department of Medicine, Imperial College London, UK; Department of Medical Statistics, London School of Hygiene and Tropical Medicine, London, UK; Huntington's Disease Research Group, Department of Neurodegenerative Disease, UCL Institute of Neurology, London, UK; Department of Psychiatry, University of Iowa, Iowa City, Iowa, USA; Department of Medical Statistics, London School of Hygiene and Tropical Medicine, London, UK; Huntington's Disease Research Group, Department of Neurodegenerative Disease, UCL Institute of Neurology, London, UK; Huntington's Disease Research Group, Department of Neurodegenerative Disease, UCL Institute of Neurology, London, UK

## Abstract

Diffusion tensor imaging (DTI) has shown microstructural abnormalities in patients with Huntington’s Disease (HD) and work is underway to characterise how these abnormalities change with disease progression. Using methods that will be applied in longitudinal research, we sought to establish the reliability of DTI in early HD patients and controls. Test-retest reliability, quantified using the intraclass correlation coefficient (ICC), was assessed using region-of-interest (ROI)-based white matter atlas and voxelwise approaches on repeat scan data from 22 participants (10 early HD, 12 controls). T1 data was used to generate further ROIs for analysis in a reduced sample of 18 participants. The results suggest that fractional anisotropy (FA) and other diffusivity metrics are generally highly reliable, with ICCs indicating considerably lower within-subject compared to between-subject variability in both HD patients and controls. Where ICC was low, particularly for the diffusivity measures in the caudate and putamen, this was partly influenced by outliers. The analysis suggests that the specific DTI methods used here are appropriate for cross-sectional research in HD, and give confidence that they can also be applied longitudinally, although this requires further investigation. An important caveat for DTI studies is that test-retest reliability may not be evenly distributed throughout the brain whereby highly anisotropic white matter regions tended to show lower relative within-subject variability than other white or grey matter regions.

## Introduction

Magnetic resonance imaging (MRI) is a valuable tool for investigating the progressive changes caused by neurodegenerative diseases, such as Huntington’s Disease (HD). A popular variant of MRI, diffusion weighted imaging (DWI) and the most commonly used analytic model, diffusion tensor imaging (DTI), can offer unique insights into the microstructural properties of both white and grey matter. Accordingly, DTI has been widely used to demonstrate regional neuroanatomical abnormalities in symptomatic HD patients 1 ^,^ 2 ^,^ 3 ^,^ 4 ^,^ 5, as well as in premanifest HD-gene carriers 6 ^,^ 7 ^,^ 8 , indicating that the technique is sensitive to some of the earliest neuropathological changes in HD.

One major strength of MRI is the ability to collect neuroanatomical data from multiple timepoints on a given sample in order to carry out longitudinal research studies; particularly pertinent in progressive diseases such as HD. Already, volumetric MRI has quantified on-going caudate nucleus atrophy across multiple disease stages 9 ^,^ 10, and offers utility as potential biomarkers of disease progression. Longitudinal DWI has been performed in HD 11 ^,^ 12 ^,^ 13 ^,^ 14, but these preliminary studies have been limited in scope and provided inconclusive results (see 15 for review).

Compared to T1- and T2-weighted imaging, DWI is prone to higher levels of image ghosting, susceptibility artefacts, eddy currents and geometric distortions, due to its reliance on single-shot echo-planar imaging 16. Crucially for movement disorders like HD, DWI is particularly sensitive to subtle bulk motion effects 17, meaning motion-induced signal loss is greater when compared with volumetric methods. These factors combine to increase the signal variability in DWI, thus it is important to demonstrate adequate test-retest (i.e. scan-rescan) reliability, if DTI is to have utility as a tool for accurately quantifying progressive microstructural changes. If within-subject variability (i.e. scan-rescan variability), influenced by the random occurrence of signal loss and distortion artefacts at different timepoints, is large relative to the between-subject variability at any one timepoint, then sensitivity to meaningful within-subject signal change will be compromised. Metrics such as intraclass correlation coefficient (ICC) can be used to quantify variability in order to provide an indication of the reliability of measurement technique.

Acceptable reliability of DTI metrics *per se* has been demonstrated previously in various settings 18 ^,^ 19 ^,^ 20 ^,^ 21 ^,^ 22 ^,^ 23 ^,^ 24, including in HD 43 ,however, what is apparent from these studies is that estimates of reliability vary considerably. Factors such as the specific image acquisition parameters, scanner characteristics (e.g. field strength, manufacturer), data processing methods or brain region under investigation 22 ^,^ 24 ^,^ 25, all lead to differing estimates of reliability. Hence the reliability of the specific techniques adopted in a longitudinal DTI study should be established, particularly if those techniques are to ever meet the exacting standards required by clinical trials; the long-term goal of MRI biomarker development. Furthermore, the presence of minor HD-related chorea in early HD patients may mean that such participants are more prone to inducing motion artefacts, reducing the signal-to-noise ratio in their acquired datasets and thus potentially leading to a problematic bias when comparing longitudinal changes with control groups.

The aim of the current study is to quantify the reliability of DTI measures in a sample of early HD patients and controls, specifically using those methods that have demonstrated cross-sectional sensitivity to HD 4 and will be used in on-going longitudinal studies. In addition, reliability will be compared between early HD patients and controls to investigate any potential disease-related group biases that may influence test-retest reliability.

## Methods

**Participants**

Ten early HD patients and 12 healthy control participants (see Table 1) were scanned at 3T (Siemens) at the Institute of Neurology, University College London. Early HD subjects were required to be within stage I of the disease 26, defined by a Unified Huntington’s Disease Rating Scale (UHDRS) Total Functional Capacity (TFC) ≥ 11, indicating good functional capacity. Control participants were spouses, partners or gene-negative siblings of the early HD subjects. Inclusion criteria included participants being over 18 years of age, free from major psychiatric and concomitant neurological disorders, not currently participating in a clinical trial and no contraindications to MRI. The local ethics committee approved the study and written informed consent was obtained from each participant. Participants were drawn from the larger PADDINGTON study (Pharmacodynamic Approaches to Demonstration of Disease-modification in Huntington's disease by SEN0014196), designed to assess potential biomarkers of HD 4. This specific subset was solely drawn from the London site where we had access to the scanner and participants were included where additional free time on the scanner was available to allow the repeated diffusion scan.

**Table 1. Demographic characteristics of early HD patients and controls:** 

Baseline characteristics	Controls		Early HD patients	
**N**	12		10	
**Age (Years)**	45.88 (15.69)	23.44-67.87	50.97 (7.68)	42.19-66.46
**Sex (male/female)**	4/8		3/7	
**CAG repeat length**			43.3 (2.4)	39 - 46
**Disease Burden [age x (cag-35.5)]**			388.8 (114.56)	232.6 - 563.4
**Total Functional Capacity**	13.0 (0.0)	13 - 13	11.5 (1.27)	9 - 13
**Total Motor Score**	0.125 (0.35)	0 - 1	22.8 (10.79)	7 - 45

Values displayed as mean (standard deviation) followed by range, for continuous variables. Discrete variables show counts of numbers. Disease burden calculated according to the formula by Penney et al., (42). Total functional capacity and total motor score are taken from the Unified Huntington’s Disease Rating Scale (UHDRS).

**MRI acquisition**

Two diffusion-weighted MRI scans were acquired at 3T for each participant using an EPI sequence with the following parameters: TR = 7600 ms, TE = 84 ms, 65 axial slices of 2 mm thickness, with no inter-slice gaps, acquisition matrix = 96 x 128, in-plane resolution of 2 mm^2^, resulting in isotropic voxels. Diffusion data were acquired in 42 different encoding directions with b = 1000 s/mm^2^, along with 7 b = 0 images. The same protocol was repeated immediately in order to acquire back-to-back datasets for test-retest reliability analysis (i.e. the participant was not removed from the scanner between acquisitions). The scanning session also included collecting a high-resolution T1-weighted MP-RAGE scan for region-of-interest (ROI) segmentation with the following parameters; TR = 2200ms, TE = 2.2ms, flip angle = 10°, FOV = 28cm, matrix size = 256x256, in-plane resolution = 1 mm^2^, slice thickness = 1.0 mm with no inter-slice gap. Visual quality controls assessed the following: compliance with relevant acquisition protocols, minimal artefacts (e.g. movement, intensity) and head positioning.

**Image analysis**

**Pre-processing**

For each participant the two DWI scans were randomly assigned into one of two independent pre-processing and statistical analysis streams. Procedures were carried out for each stream separately. This was done to ensure no effects of acquisition order influenced the results. Firstly, diffusion-weighted images were registered to the mean of the seven b0 images to correct for motion and eddy current distortions, and the gradient direction scheme was updated accordingly. Subsequently, a non-linear least-squares method was used to fit the tensor at each voxel. Scalar maps of diffusion metrics such as fractional anisotropy (FA), mean (MD), axial (AD) and radial diffusivity (RD) were then derived from these tensor images. In order to carry out a comprehensive assessment of test-retest reliability, three different approaches were used: a T1 ROI analysis (as per 4) where the analysis was conducted in native diffusion space, an atlas-based automated white matter ROI analysis and a voxelwise analysis.

**T1 ROI analysis**

Four ROIs were defined on the T1 images. For the caudate, corpus callosum and cerebral white matter regions manual delineation was carried out using the MIDAS software package 27. For the putamen the automated BRAINS3 program was used 28. The resulting ROIs were transformed into native diffusion space by first registering the T1 image to the participant’s FA image, using an initial affine registration, followed by a non-linear registration, necessary to account for the non-linear distortions found in DWI. This was achieved using Nifty-Reg (http://sourceforge.net/projects/niftyreg) for both the affine 29 and non-linear 30 stages. The transformation from T1 to native FA space was then applied to the binary ROI labels using a nearest neighbour interpolation scheme. Registration accuracy for all data was assessed visually to ensure accurate placement of ROIs in diffusion space. The mean FA, MD, AD and RD values across the corpus callosum, cerebral white matter and bilateral caudate and putamen ROIs was then calculated using FSL (http://fsl.fmrib.ox.ac.uk). Four control participants did not have T1-weighted scans and were excluded from this element of the analysis, leaving 10 early HD patients and 8 controls with data for the T1 ROIs.

**Automated atlas-based ROI analysis**

Tensor images were converted into DTI-TK format (http://dti-tk.sourceforge.net) to run tensor-based registration, a method shown to improve registration accuracy compared with using FA images 31. Using the standard DTI-TK pipeline, a ‘bootstrap’ template was defined by an affine registration step to put all subjects into approximately the same space. Each native-space tensor image was non-linearly aligned to this template, using an iterative approach to refine the accuracy of the registration until the difference between successive iterations becomes minimal, based on the Euclidean distance of the tensors 32. Affine and non-linear transformation parameters were combined to allow the native space tensor images to be warped to common space in a single interpolation step. Once all the images were in a common space, the mean FA map was generated to act as a study specific template. As mentioned above, this was done independently for both processing streams; hence two separate templates were produced.

With all the tensor images aligned to the group template, FA, MD, AD and RD maps were generated for each participant. The next stage was to take the white matter ROIs defined by the ICBM-DTI-81 white matter tract atlas 33, which is supplied with FSL and contains labels for 48 white matter regions. The 2mm^3^ ICBM-DTI-81 atlas image was registered to the group FA template using Nifty-Reg to run an initial affine step followed by a non-linear refinement stage. The resultant transformation was then used to warp the white matter label files to group FA template space, which were finally thresholded at template FA > 0.2 to reduce partial volume effects. Mean FA, MD, AD and RD were then computed across each label region using FSL. This was repeated for the second processing stream and the resultant values were used in the subsequent reliability analyses.

**Statistical analysis**

As a simple assessment of agreement, Bland-Altman plots 34 were used to examine variability of DTI metrics within each T1 ROI, with HD patient and control data combined.

A common measure of reliability, the intraclass correlation coefficient (ICC) was used to assess the reliability of the scanning procedure in greater detail, and was calculated for controls and HD patients separately, for each region. Confidence intervals (CIs) at 95% were obtained for ICC values using the delta method. The within- and between-subject variances were also calculated for each measure. ICCs were unadjusted for age and sex in order to avoid making the methods incomparable with previous studies of DTI reliability 22 ^,^ 24.

**Voxelwise analysis**

A similar procedure to the atlas-based analysis was used, with the addition of a within-subject registration step, in order to increase the likelihood of voxelwise correspondence across the brain. Again using DTI-TK to register the tensor images, scans from both processing streams for each participant were co-registered using the initial affine and iterative non-linear steps as detailed above. Once co-registered to an unbiased ‘mid-space’, the subject means were calculated and then fed into the registration pipeline to define a group template, this time combining scans for both processing streams. The transformations from the separate registration steps were then combined together to allow registration from native space to this combined group space in one interpolation step. The ICC could then be calculated for both FA and MD at each voxel using the* fslmaths* utility in FSL. This registration procedure and subsequent statistical analysis was first completed using the 10 early HD patients and the using data from the 12 control participants.

## Results

**Regional reliability analysis**

Test-retest reliability of T1-based and atlas ROIs showed generally high ICCs indicating good of levels of reliability (Tables 2-5). This was the case for all diffusion metrics (i.e. FA, MD, RD and AD). However, some variability in reliability was present across different brain regions.

**Figure:**
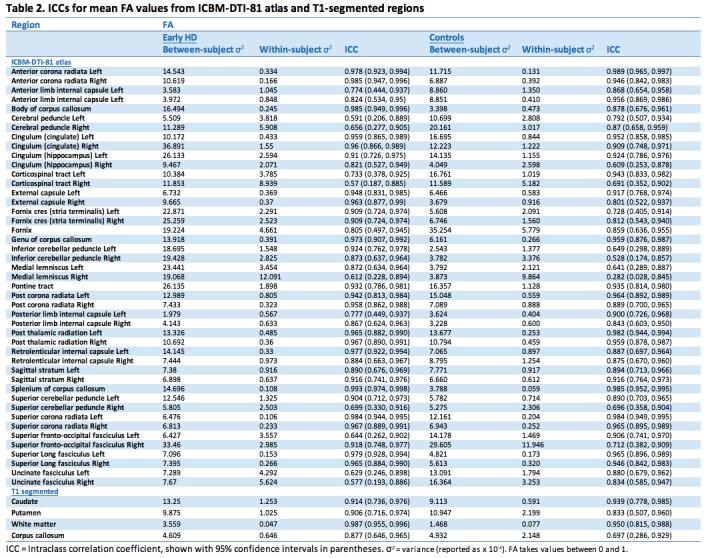

**Figure:**
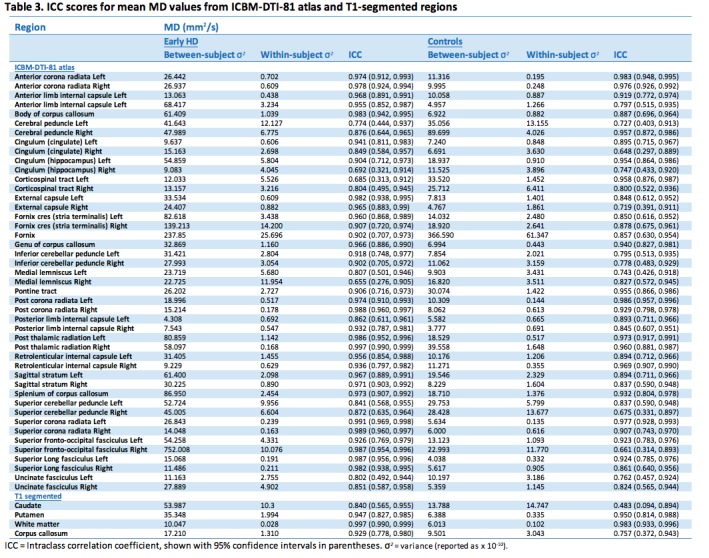

**Figure:**
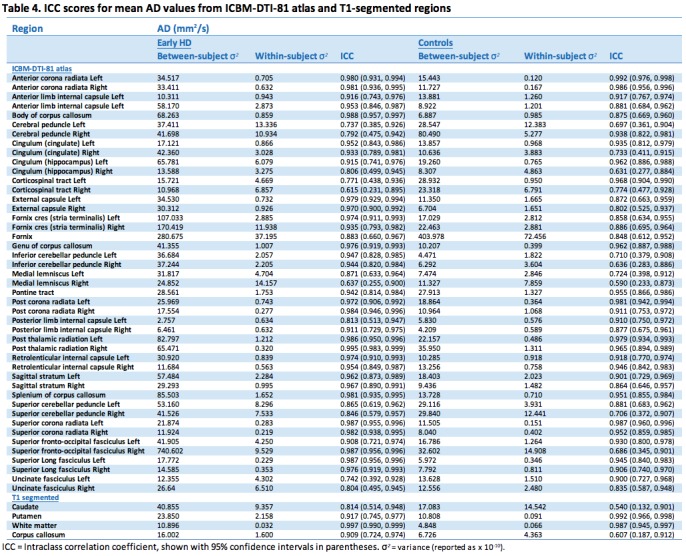

**Figure:**
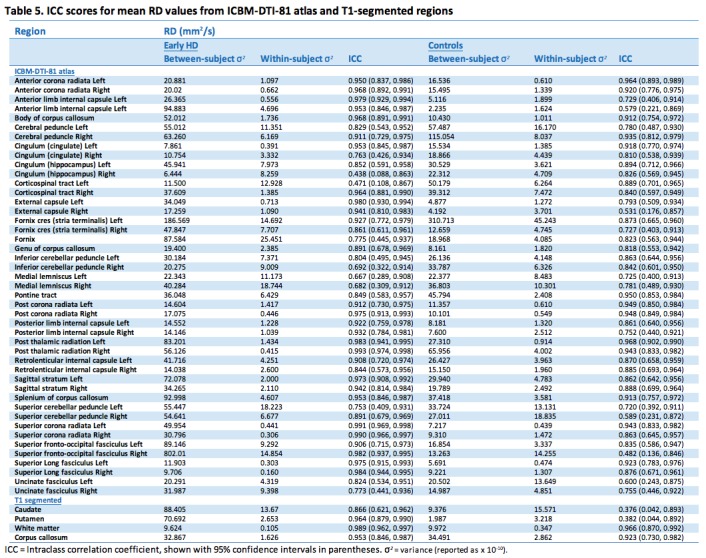

For FA in the controls, 38 (79%) atlas ROIs had an ICC of 0.8 or above, with the majority of these being > 0.9. Exceptions to this included the corticospinal tract and cerebellar ROIs. This pattern was reflected in the early HD patients, with 41 (85%) ROIs having ICCs > 0.8. Generally, between-subject variance was larger in the HD group than controls, as would be expected. It is also worth noting that the within-subject variances are relatively similar for controls and HD in many regions. This implies that, in general, differences in ICC observed between controls and HD are driven by the between-subject variation rather than by the scanning technique being less reliable in one group than the other.

For MD 37 (77%) atlas ROIs had ICCs above 0.8 in the controls; the corresponding number was 44 (92%) in the early HD patients. As with FA, any between group differences in ICC were accompanied by wide 95% CIs around each group estimate, demonstrating the imprecision in the estimates. Results from AD were similar, where 33 (69%) atlas ROIs had ICCs > 0.8, and RD with 37 (77%) ROIs having ICCs > 0.8. In controls mean ICCs were very similar across metrics (FA mean ICC = 0.851; MD = 0.854; AD = 0.811; RD = 0.857). However, a few ROIs did show discrepancies between metrics, such as the right anterior limb of internal capsule and right external capsule, which exhibited substantially lower ICCs in AD compared to the other metrics. This is driven by the much smaller between-subject variation for AD than other for metrics in the right anterior limb of internal capsule, and by an increased within-subject variability for the external capsule. Examination of scatter plots of the data identified two outliers that may have contributed to this.

For the T1 ROIs, FA reliability was also high, with the caudate, putamen and whole-brain white matter regions having ICCs > 0.8 in controls and early HD patients. The corpus callosum region had a lower ICC in the controls (ICC = 0.697) though the ICC was high in the early HD patients (ICC = 0.877). For MD, there was considerable regional variability in reliability, with very high ICCs for the putamen and white matter (ICC > 0.95), an intermediate value for the corpus callosum (ICC = 0.76) and a low value for the caudate (ICC = 0.48). Furthermore, the ICC in the early HD patients was reasonably high for caudate MD (ICC = 0.84). For AD, both the caudate and the putamen had low reliability in the controls (ICCs < 0.4), though for RD the putamen values were very high (ICC = 0.99). The corpus callosum ROI showed the converse pattern, with a very high ICC for AD (ICC = 0.91) and a low one for RD (ICC = 0.61). The white matter ROI showed very high reliability (ICCs > 0.9) across the board.

To put these divergent results from the T1 ROIs into context, the Bland-Altman (BA) plots were considered for FA (Figure 1) and MD (Figure 2). These generally suggest good agreement, with the differences tending to lie within a small range. The exceptions to this were MD in the caudate, and FA and MD in the corpus callosum. In these cases, there was a suggestion of deviation from exact reproducibility in the combined HD patient and control group. However, the BA plots do not suggest that the difference between scans is dependent on the magnitude of the measurement in question, and indicated the possibility of outliers within the data.

**Bland-Altman plot for FA values:**
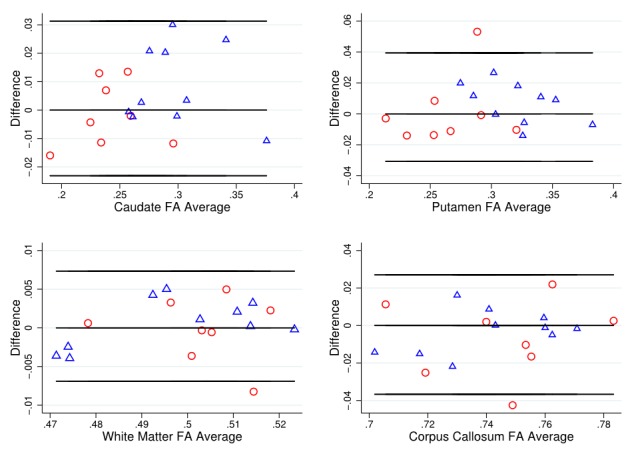
Bland Altman plots of fractional anisotropy (FA) values to visually assess agreement, systematic bias and proportional bias in scanning technique for T1 ROIs (caudate, putamen, white matter and corpus callosum), for early HD patients (blue triangles) and controls (red circles). FA is a relative value derived from the diffusion tensor, where 0 indicates perfectly isotropic tensor dimensions (i.e. a sphere) and 1 indicates the maximum theoretical level of anisotropy.

**Bland-Altman plot for MD values:**
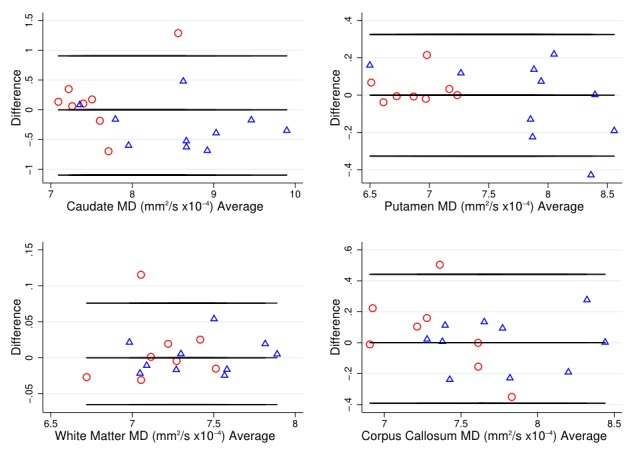
Bland Altman plots of mean diffusivity (MD) values to visually assess agreement, systematic bias and proportional bias in scanning technique for T1 ROIs (caudate, putamen, white matter and corpus callosum), for early HD patients (blue triangles) and controls (red circles).

**Bland-Altman plot for AD values:**
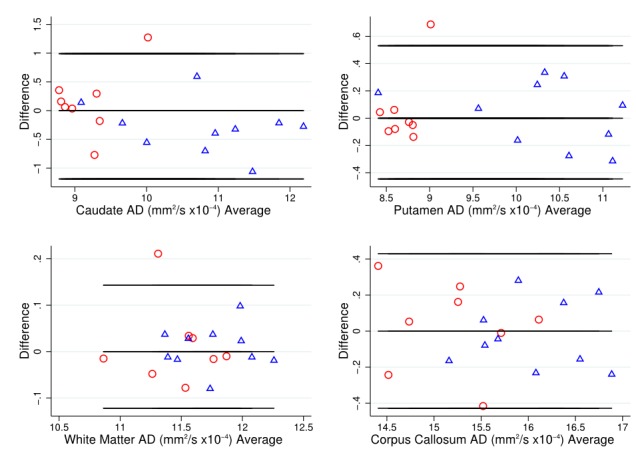
Bland Altman plots of axial diffusivity (AD) values to visually assess agreement, systematic bias and proportional bias in scanning technique for T1 ROIs (caudate, putamen, white matter and corpus callosum), for early HD patients (blue triangles) and controls (red circles).

**Bland-Altman plot for RD values:**
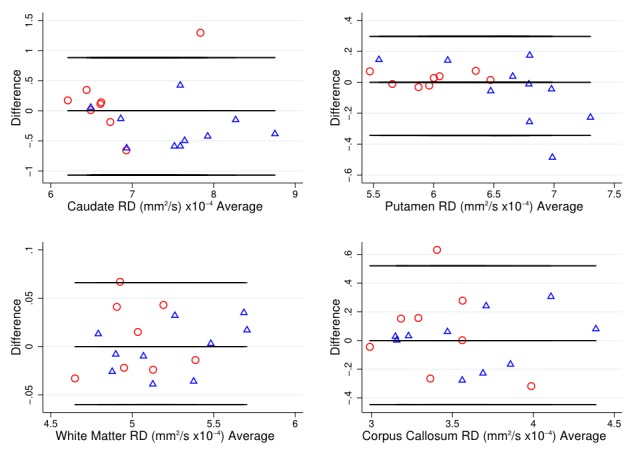
Bland Altman plots of radial diffusivity (RD) values to visually assess agreement, systematic bias and proportional bias in scanning technique for T1 ROIs (caudate, putamen, white matter and corpus callosum), for early HD patients (blue triangles) and controls (red circles).

**Voxelwise reliability analysis**

The ICC maps for FA (Figure 3), MD (Figure 4), AD (Figure 5) and RD (Figure 6) indicated that the reliability was generally high across the brain, with the exception of areas inferior to the lateral ventricles. There is also a degree of noise evident in these maps, likely due to residual registration error between voxels. The group maps appeared qualitatively similar between controls and early HD patients. FA and MD also seemed to generate similar patterns of voxelwise reliability, although the diffusivity metrics (i.e. MD, AD, RD) tend to show consistently higher ICC scores with less regional variability than FA. Lower ICCs were evident in the basal ganglia regions for all four measures.

**Voxelwise ICC maps of FA:**
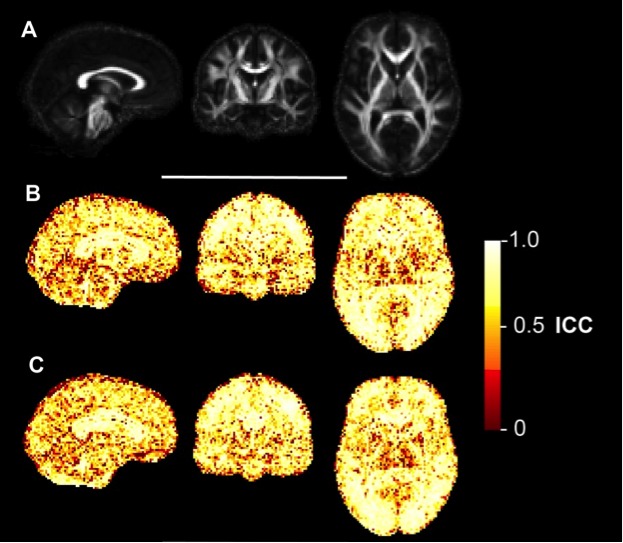
Voxelwise distribution of reliability metrics for fractional anisotropy (FA). Panel A) shows sagittal, coronal and axial slices of the mean FA image created during the image processing, included for anatomical reference. B) Equivalent three slices for the intraclass correlation coefficient (ICC) of FA in early HD patients. Higher values reflect higher test-retest reliability. C) ICC of FA in healthy controls.

**Voxelwise ICC maps for MD:**
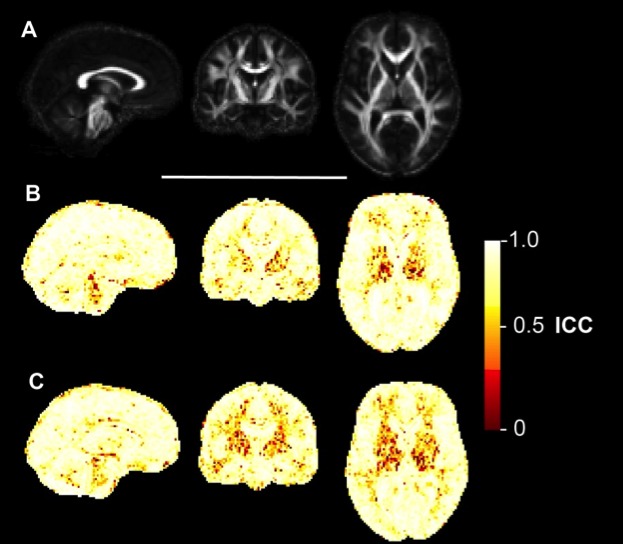
Voxelwise distribution of reliability metrics for mean diffusivity (MD). Panel A) shows sagittal, coronal and axial slices of the mean FA image created during the image processing, included for anatomical reference. B) Equivalent three slices for the intraclass correlation coefficient (ICC) of MD in early HD patients. Higher values reflect higher test-retest reliability. C) ICC of MD in healthy controls.

**Voxelwise ICC maps of AD:**
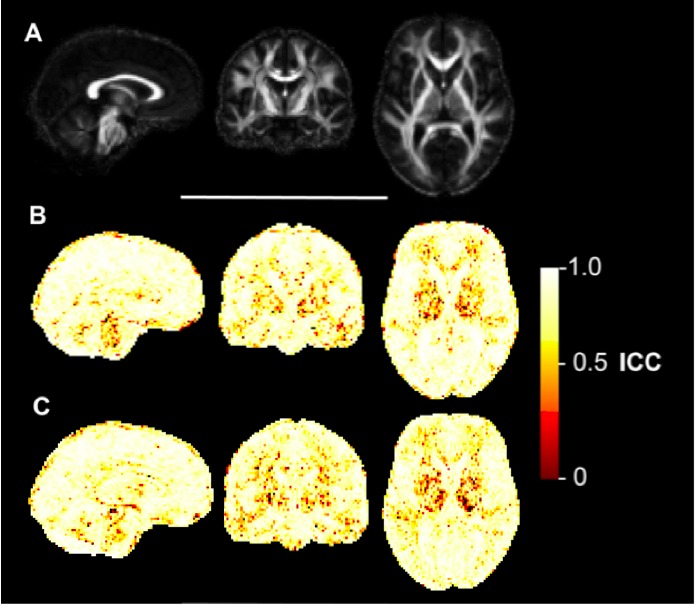
Voxelwise distribution of reliability metrics for axial diffusivity (AD). Panel A) shows sagittal, coronal and axial slices of the mean FA image created during the image processing, included for anatomical reference. B) Equivalent three slices for the intraclass correlation coefficient (ICC) of AD in early HD patients. Higher values reflect higher test-retest reliability. C) ICC of AD in healthy controls.

**Voxelwise ICC maps of RD:**
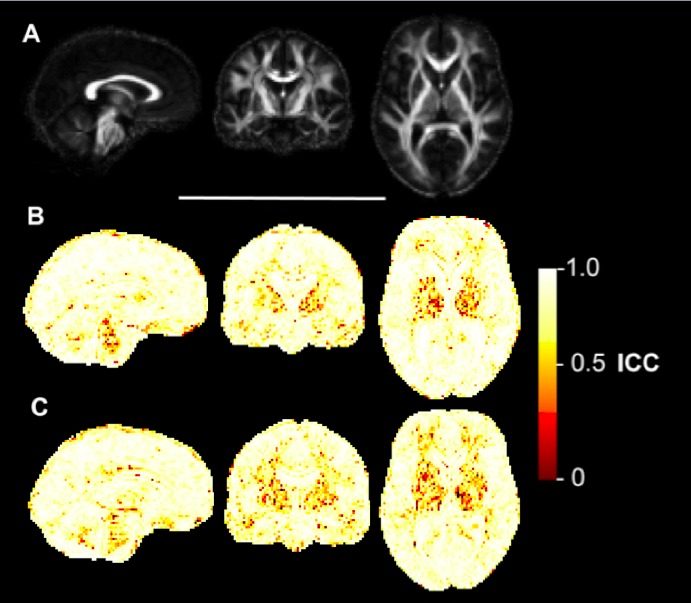
Voxelwise distribution of reliability metrics for radial diffusivity (RD). Panel A) shows sagittal, coronal and axial slices of the mean FA image created during the image processing, included for anatomical reference. B) Equivalent three slices for the intraclass correlation coefficient (ICC) of RD in early HD patients. Higher values reflect higher test-retest reliability. C) ICC of RD in healthy controls.

## Discussion

Analysis of DTI scans acquired back-to-back gave generally high levels of reliability in both early stage HD patients and controls, when using either atlas-based or T1-segmentation-based ROIs. Using specific methods that will be applied to longitudinal datasets in studies of HD, the four major DTI metrics (FA, MD, AD and RD) all showed low levels of within-subject (i.e. scan-rescan) variability relative to between-subject variability. This concurs with previous research that has assessed the reliability of DTI in HD 43 and other samples using different analysis methods, which generally report acceptable levels of reliability (i.e. high ICC), either test-retest 24 ^,^ 35 or between-scanners 21 ^,^ 22 ^,^ 36 ^,^ 37. All metrics tended to perform as well in both experimental groups and importantly there was no qualitative evidence of group bias in reliability. Although no formal statistical comparisons were made between HD and controls, the ICC estimates and 95% CI suggested that for most regions the ICCs were consistent between groups. Given the magnitude of DTI effect sizes in cross-sectional comparisons of early HD patients and controls (e.g. 4), the low within-subject variances found support the continued use of DTI as a tool for detecting patterns of neurodegeneration in this HD population.

It is important to recognise the imprecision in the ICCs reflected in the relatively wide confidence intervals, particularly for the lower ICCS. Nonetheless it does appear that levels of reliability were not entirely consistent across the brain. Those regions that showed lower reliability (ICC < 0.8) tended to be regions in the inferior of the brain, including cerebellum and brainstem ROIs. Explanations for this could include the increased presence of tissue susceptibility errors in inferior regions, or sub-optimal image registration due to the registration procedure being largely driven by the increased contrast in signal intensity found in and around the lateral ventricles 32. Also, these cerebellar regions have smaller volumes when defined according to the ICBM-DTI-81 atlas, thus may be more susceptible to partial volume effects than larger areas where such effects are more likely to be averaged out. The present findings indicate that extra caution should be exercised when examining patterns of longitudinal change in small inferior regions, as these tend to have greater inherent variability using atlas-based registration methods.

Interestingly, the grey matter ROIs, segmented on a T1 image, showed lower reliability for diffusion metrics, particularly MD in the caudate and AD in both caudate and putamen. Regional atrophy may contribute to inaccuracies in the registration of T1 images to FA maps and the caudate in particular is susceptible to partial volume effects due to its proximity to the lateral ventricles. The process of transforming T1 ROIs to diffusion space is performed regularly in DTI ROI analyses and although not routinely reported, visual quality assurance of registration accuracy is absolutely necessary as achieving precise anatomical correspondence between images acquired using different modalities is challenging.

Although the low ICC values in the striatal ROIs may be some cause for concern for longitudinal studies, this may be explained by the presence of particularly low between-subject variability, meaning that minor divergences within a small number of participants can lead to large fluctuations of the estimated ICC. The influence of such outliers, particularly in light of the reduced control sample size for the T1-segmented regions (N = 8), could be pronounced. To assess this, outliers identified from scatter plots were removed and the ICCs recalculated. In most cases, the ICC increased on removal, though not necessarily to the high values observed for other regions. As an example, AD in the left corticospinal tract had a low ICC in the HD subjects. Removing a single outlier increased the ICC from 0.471 to 0.763, which is still lower than the value of 0.889 observed for the same region in the controls, or 0.964, found in HD patients in the right hemisphere.

The within-subject variance should also be considered when comparing between regions or metrics. While in general within-variance was strongly related to ICC, as would be expected, there are some regions, such as the fornix, where ICC was high but within-subject variance appeared much greater than other regions. The magnitude of the within-subject variance is approximately inversely proportional to the amount of signal, so for example, one may be cautious about the reliability of the fornix for longitudinal studies, despite a relatively high ICC of 0.90 for MD. If wishing to restrict the number of brain regions tested, areas such as the genu and body of corpus callosum, for which we observed high ICC and very low within-subject variance, might be more appropriate for longitudinal research studies.

Voxelwise ICC maps of FA, MD, AD and RD indicated that the distribution of variability was qualitatively very similar between early HD patients and controls. High ICC for FA was apparent across the white matter of the brain, with reduced values in the lateral ventricles and subcortical grey matter nuclei. The strength of the signal in DTI depends on the degree of anisotropy 38 and FA is a direct representation of this measure, so it is plausible that the inherent anisotropy of brain tissue influences the between-scan variability. The MD ICC maps also did not materially differ between groups, though when compared with the FA maps, the pattern was one of generally higher ICC throughout the brain, which was matched by very similar patterns for AD and RD; not unexpected given the relatedness of the metrics. The voxelwise patterns of reliability concur with the study by Marenco and colleagues 24 in healthy controls at 1.5T who showed a similar distribution of ICC for FA and trace (i.e. total diffusivity). In accordance with this, the voxelwise and ROI-based findings may add weight to the idea that DTI in general, and FA in particular, is primarily suited for examining white matter microstructure 22 and is less quantifiable 39 and harder to interpret in grey-matter regions 40. Measures of anisotropy in tissue not thought to be characteristically anisotropic may not give particularly meaningful insight into biological or pathological processes.

Although the four DTI metrics were generally comparable across the board, there was a trend for lower reliability in AD, compared with FA, MD or RD. One possible explanation for this finding is that AD, unlike any of the other metrics, is derived from a single component of the diffusion tensor. For FA, MD and RD there is a degree of averaging across tensor elements (i.e. eigenvalues) that may increase the signal-to-noise ratio and reduce variability, whereas AD is derived solely from the primary eigenvalue. Previous reliability studies have not reported on AD before and this potential difference with other diffusion metrics should be investigated further, particularly if AD results are to be analysed longitudinally

There are a few caveats to consider when interpreting the present findings. The sample size was relatively small once divided into early HD patients and controls, thus resulting in increased susceptibility to the influence of outliers and reduced precision. One issue in extrapolating these findings to future longitudinal studies is the scan-rescan data was collected back-to-back, with the participant remaining in the scanner. This meant that the position of the head within the magnetic field was very similar between scans, which is less often the case with longer intervals where re-positioning is required. When running longitudinal studies, monitoring the consistency of head positioning at each acquisition could help reduce the variability that can be caused by subtle differences in orientation and slice positioning 19 ^,^ 41 . Another limitation is that these results are scanner specific. The reliability of DTI may differ between scanner manufacturers 37, or show higher noise/within-subject variability in older machines that require servicing. This point is particularly relevant to multi-centre studies, which are likely to be increasingly necessary in relatively uncommon diseases such as HD, in order to have well-powered studies. While the reliability of DTI has been demonstrated across scanners in principle 36, collecting repeat scans both within-scanner and across study sites would be a prudent step to help establish the reliability of DTI data in any large scale study.

In conclusion, test-retest reliability analysis of atlas-based and T1-segmented ROI approaches DTI analysis show generally high ICCs on diffusion data acquired with a clinically-acceptable scan time. This gives confidence that such data acquisition and analysis methods can be reliably used for cross-sectional comparisons, alongside lending support for their utility to measure within-subject change over time; the goal of on-going longitudinal research into progressive neurodegeneration in HD. However, longitudinal reliability can only be explicitly demonstrated by taking the expected magnitude of the longitudinal effects into account, to determine whether these effects are greater than the scan-rescan variability. Finally, it is notable that there are some inconsistencies to the generally high reliability; particularly in striatal AD measures and it could be concluded that test-retest reliability is not evenly distributed throughout the brain, potentially due to intrinsic tissue differences, non-linear geometric distortions or uneven registration accuracy. This has implications for selecting which brain regions are most appropriate for future longitudinal studies, above and beyond the biological evidence for involvement in neurodegeneration.

## Competing Interests

The authors have declared that no competing interests exist.
